# Disparities in Sources of Added Sugars and High Glycemic Index Foods in Diets of US Children, 2011–2016

**DOI:** 10.5888/pcd17.200091

**Published:** 2020-11-05

**Authors:** Rienna G. Russo, Brandilyn A. Peters, Vanessa Salcedo, Vivian HC Wang, Simona C. Kwon, Bei Wu, Stella Yi

**Affiliations:** 1 New York University, School of Medicine, Department of Population Health, New York, New York; 2 Albert Einstein College of Medicine, Department of Epidemiology and Population Health, Bronx, New York; 3 New York University, School of Global Public Health, New York, New York; 4 New York University, Rory Meyers College of Nursing, New York, New York

## Abstract

**Introduction:**

Added sugars and high glycemic index (GI) foods might play a role in cardiometabolic pathogenesis. Our study aimed to describe the top sources of added sugars and types of high GI foods in diets of children by race/ethnicity.

**Methods:**

We examined data for 3,112 children, aged 6 to 11 years from the National Health and Nutrition Examination Survey (NHANES), 2011 to 2016. Mean intake was estimated and linear regression models tested for differences by race/ethnicity. Population proportions for food sources were created and ranked, accounting for survey weighting when appropriate.

**Results:**

Asian American and Mexican American children had the lowest reported added sugar intake. Cereals were observed to contribute highly to added sugar intake. Soft drinks did not contribute as much added sugar intake for Asian American children as it did for children of other races/ethnicities. Asian American children consumed significantly more high GI foods than other groups. Types of high GI foods differed meaningfully across racial/ethnic groups (ie, Mexican American: burritos/tacos; other Hispanic, White, and Black: pizza; Asian American: rice). Rice accounted for 37% of total high GI foods consumed by Asian American children.

**Conclusions:**

Sources of added sugars and types of high GI foods in children’s diets vary across racial/ethnic groups. Targeting foods identified as top sources of added sugars for all race/ethnicities and focusing on substitution of whole grains may reduce obesity, diabetes, and related cardiometabolic risk more equitably.

**Summary**

**What is already known on this topic?**

Added sugars and high glycemic index (GI) foods may contribute to the development of cardiometabolic conditions. No studies have investigated whether or not there are racial/ethnic differences in the consumption of top sources of added sugars or high GI foods among children.

**What is addressed by this article?**

Our study determined whether the consumption of top sources of added sugars and high GI foods among children in the United States differed by race/ethnicity.

**What are the implications for public health practice?**

Products with hidden added sugars contribute greatly to added sugar intake. High GI food intake was highest among Asian Americans for whom rice is a staple. Findings underscore the need to account for sociocultural differences when creating dietary modification strategies.

## Introduction

Childhood obesity remains a challenge, despite numerous policies and programs focused on improving nutrition and increasing physical activity among children (1). Disparities in obesity, diet, and physical activity are clear for Hispanic and Asian American children, but data are limited (2–4). According to national and local estimates, the burden of obesity is highest for Hispanic children in the United States. Although obesity prevalence is lowest among Asian American children (3), anthropomorphic differences (eg, high percentage body fat, low muscle mass) among Asian populations have led to the broad consensus that current definitions of overweight and obesity likely underestimate the true burden of the metabolic effects of obesity among Asian American children (5). Evidence is mounting about the long-term vulnerability of these children; Hispanic and Asian American children are at the highest risk for nonalcoholic fatty liver disease (6); therefore, they are at greater risk than children of other races/ethnicities for cardiometabolic problems throughout the life course. Yet, few studies have focused on these population subgroups.

Studies have also recognized that the top dietary sources of sodium and preferred types of beverages for Hispanic and Asian Americans are different from those for non-Hispanic White and Black Americans (4,7), despite that most nutrition policies and programs target non-Hispanic White and Black Americans. This implies a mismatch of initiatives to improve nutrition for Hispanic and Asian American children. Incompatible cultural policies and programs, lack of attention to equitable implementation, and prolonged disparate funding will lead to greater disparities in obesity and nutrition over time for Hispanic and Asian American children (8,9).

Added sugars have been implicated as a leading predictor of dietary cardiometabolic concerns amongst children, including obesity, diabetes, and nonalcoholic fatty liver disease (10–12). Added sugars do not include naturally occurring sugars, such as lactose in milk and fructose in 100% fruit juice (13). Refined grains and other foods with a high glycemic index (GI) might also contribute to cardiometabolic conditions, because insulin resistance is involved in future diabetes and development of nonalcoholic fatty liver disease. High glycemic load results in increased risk of insulin dysregulation as well. Excess weight gain, elevated blood pressure, and type 2 diabetes all share links to added sugar and consumption of high GI food (11,14–16).

The leading source of added sugars in children’s diets is often sugar-sweetened beverages; however, no research has investigated other sources of added sugars that are most often consumed by racial/ethnic groups. Additionally, refined grains and other high GI foods might play a significant role in diabetes and the development of nonalcoholic fatty liver disease, especially among Asian American and other Hispanic (non-Mexican) populations for whom rice is traditionally a staple food (17). Because these are potential food sources of cardiometabolic risk among children, our study aimed to 1) examine racial/ethnic differences in amounts of calories and added sugars consumed, 2) examine high GI food intake, 3) identify the top 10 sources of added sugars and types of high GI foods, and 4) stratify findings by race/ethnicity.

## Methods

The National Health and Nutrition Examination Survey (NHANES) is a cross-sectional study designed to assess the health and nutrition of the US population. NHANES uses a stratified, multistage probability sampling design to recruit a nationally representative sample of participants. Participant data are collected through in-person household interviews and follow-up health examinations each year, then released in a series of 2-year cycles. Details of the survey, including its content and operations, are publicly available (https://www.cdc.gov/nchs/nhanes/index.htm). Data from 2011 through 2016 were used in our study, because Asian Americans were oversampled during these cycles (https://www.cdc.gov/nchs/data/series/sr_02/sr02_162.pdf). Children aged 6 to 11 years were included in the study if they had a reliable and complete first of 2 days of dietary recalls.

Sociodemographic characteristics used to describe the sample were self-reported during in-home interviews. Variables included race/ethnicity (Mexican American, other Hispanic, non-Hispanic White, non-Hispanic Black, non-Hispanic Asian American, and non-Hispanic other), sex, nativity (US-born or foreign-born), and poverty index ratio (< 1.0, 1.0–3.0, >3.0) — a measure based on the federal poverty threshold set by the US Census Bureau (18). Data for Hispanic and Asian American subgroups were unavailable because of limited sample sizes. Poverty index ratio and nativity were also used as covariates in regression analyses when assessing differences in mean added sugars and high GI intake by race/ethnicity.

Individual food and beverage items were linked with What We Eat in America (WWEIA) categories, a classification system for foods and beverages released every 2 years (19). We aligned the coding of categories in the 2011–2012 and 2013–2014 WWEIA cycles with the 2015–2016 cycles to account for any changes in the classification scheme.

High GI foods were identified by using the 2008 International Tables of Glycemic Index and Glycemic Load Values (20), and the most common foods were included in the index. Foods were assigned a WWEIA category, on the basis of their description and nutritional content, and average GI and corresponding WWEIA categories were compared.

We estimated means and SDs for energy in calories and added sugars in grams (g). We also estimated percentages of total calories and grams consumed from the high GI foods accounted. Multivariate linear regression models were used to compare mean intake of dietary components by race/ethnicity, adjusting for poverty and nativity, because these characteristics varied among races/ethnicities. Regression coefficients (β) and corresponding 95% CIs were obtained.

Percentages of population were calculated for added sugars and total GI foods by summing the amount consumed in each food category for participants in each racial/ethnic group and dividing the sum by the total amount for all persons in that racial/ethnic group, then multiplying by 100%. The population proportions were then ranked to determine the top 10 food sources of added sugars and types of high GI foods.

We used Stata version 15 (StataCorp LLC) to stratify analyses by race/ethnicity. Stata’s SVY commands were used to account for the complex sampling design and weighting. A 6-year dietary weight was generated by using one-third of the 1-day dietary weight for each 2-year cycle.

## Results

A total of 3,112 children were included in our sample. About 1,618 children were White (52%), and 809 (26%) lived below the federal poverty threshold. Most children were US-born (2,939 children, 96%). Some differences by race/ethnicity were observed. Compared with nearly one-half of Mexican American children (268/605, 45%) and Black children (355/779, 46%), only 1 in 7 White (180/799, 15%) and Asian American (31/220, 13%) children were below the poverty threshold. Except for Asian American children, more than 90% of all other racial/ethnic subgroups were born in the United States (Mexican American, 605/659; other Hispanic, 333/365; White, 812/820; Black, 804/823; Other, 198/204). Approximately three-fourths (187/241, 78%) of Asian American children were born in the United States (Table 1).

**Table 1:** Demographic Characteristics of Children Aged 6–11 Years (N = 3,112), by Race and Ethnicity, NHANES 2011–2016^a^

Characteristic	Mexican American	Other Hispanic	White	Black	Asian American	Other	Total	*P *Value
**Sex**								
Male	48.2 (42.4–54.1)	58.3 (52.6–63.8)	54.4 (49.3–59.5)	52.6 (48.4–56.8)	53.3 (44.8–61.7)	43.8 (32.1–56.2)	52.9 (49.6–56.2)	.12
Female	51.8 (45.9–57.6)	41.7 (36.2–47.4)	45.6 (40.5–50.8)	47.4 (43.2–51.6)	46.7 (38.3–55.2)	56.24 (43.8–68.0)	47.1 (43.9–50.36)	
**Poverty ratio^b^**								
<1.0	44.8 (38.8–50.8)	39.6 (32.0–47.6)	14.2 (11.1–18.0)	46.3 (41.2–51.4)	13.3 (6.9–23.9)	25.9 (18.2–35.6)	26.0 (22.5–29.8)	<.001
1.0–3.0	43.1 (37.9–48.5)	39.7 (32.6–47.3)	38.6 (32.8–44.7)	42.0 (37.4–46.7)	32.0 (23.2–42.6)	37.1 (26.9–48.7)	39.5 (35.8–43.4)	
>3.0	12.1 (7.9–18.2)	20.75 (15.2–27.7)	47.2 (39.8–54.8)	11.7 (9.1–15.0)	54.7 (43.2–65.7)	37.0 (26.2–49.1)	34.5(29.2–40.3)	
**Nativity**								
US-born	92.8 (89.0–95.4)	91.6 (88.2–94.0)	99.3 (98.1–99.8)	98.0 (95.8–99.1)	77.2 (71.4–82.1)	96.4 (88.7–98.9)	96.3 (95.2–97.2)	<.001
Non-US–born	7.2 (4.7–11.1)	8.5 (6.0–11.8)	0.7 (0.3–1.9)	2.0 (0.9–4.2)	22.8 (17.9–28.6)	3.7 (1.1–11.4)	3.7 (2.8–4.8)	

Abbreviation: NHANES, National Health and Nutrition Examination Survey.

a Values are percentage (95% CI).

b Measures based on the federal poverty threshold set by the US Census Bureau.

Mean added sugar consumption was lowest among Mexican American children (mean 21.8 g, SD 22.6) and Asian American children (mean 25.0 g, SD 28.8) and highest among White children (mean 31.9 g, SD 18.6). We observed no significant differences in mean total calories (Figure). No significant differences were observed in caloric intake between Asian American children and other races/ethnicities. Mexican American children consumed significantly less added sugars (−3.2 g, *P* < .05) and White children consumed significantly more (6.9 g, *P* < .001) than Asian American children.

**Figure:**
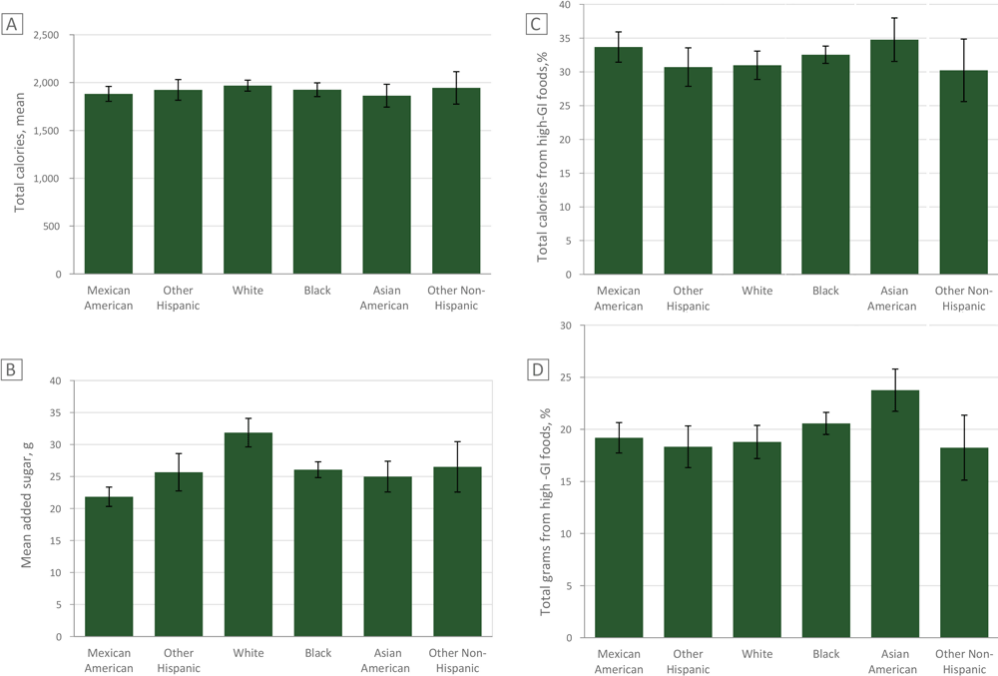
Mean added sugars consumed by children, by race/ethnicity, and proportion of calories and grams consumed from high glycemic index (GI) foods. Values are based on data from the National Health and Nutrition Examination Survey 2011–2016 (21). Figure A: Comparison of total kilocalories consumed. Figure B. Comparison of total grams of added sugars consumed. Figure C: Comparison of percentage total calories consumed from high glycemic index (GI) foods by race/ethnicity. Figure D: Comparison of percentage total grams consumed from high GI foods.

**Table:** 

Race/Ethnicity	A. Total Kilocalories Consumed, Mean (95% CI)	B. Added Sugar Consumed, Mean (95% CI)	C. Total Added Kilocalories of High GI Foods Consumed, % (95% CI)	D. Total Grams of Added High GI Foods Consumed, % (95% CI)
Mexican American	1,882.0 (1,804.0–1,960.1)	21.8 (20.3–23.4)	33.7 (31.5–35.9)	19.2 (17.7–20.8)
Other Hispanic	1,923.8 (1,815.8–2,031.8)	25.67 (22.7–28.6)	30.71 (27.9–33.6)	18.3 (16.3–20.3)
White	1,968.7 (1,911.7–2,025.7)	31.9 (29.6–34.1)	30.9 (28.8–33.1)	18.8 (17.2–20.4)
Black	1,925.6 (1,853.81–1,997.3)	26.1 (24.9–27.3)	32.5 (31.3–33.8)	20.6 (19.52–21.6)
Asian Americans	1,863.3 (1,744.10–1,982.5)	24.0 (22.6–27.4)	34.8 (31.6–38.0)	23.8 (21.73–25.8)
Other Non-Hispanic	1,945.2 (1,775.8–2,114.6)	26.5 (22.6–30.5)	30.2 (25.6–34.9)	18.2 (15.1–21.4)

Candy not containing chocolate was the leading source of added sugars for children among all racial/ethnic subgroups, except for other Hispanic children, whose top source was high-sugar cereal. Baked goods and breakfast foods (eg, ready-to-eat cereal, doughnuts, jams, syrups) appeared most frequently in the top 10 sources of added sugar. For Mexican American and Black children, soft drinks and sweetened fruit drinks were among the top sources, whereas for other Hispanic, Asian American, and other racial/ethnic children only sweetened fruit drinks were among the top sources. For White children, only soft drinks were among the top sources. We identified the top 10 sources of added sugars consumed by children aged 6–11 (Table 2).

**Table 2:** Ranked Population Proportion of 10 Added Sugars Consumed by Children Aged 6–11 Years (N = 3,112), by Race and Ethnicity, NHANES 2011–2016^a^

Race/Ethnicity, Rank	Food Source	Percentage	Cumulative Percentage^b^
**Mexican American**			
1	Burritos and tacos	24.9	24.9
2	Tortillas	11.1	35.9
3	Egg/breakfast sandwiches	7.0	42.9
4	Pizza	6.9	49.8
5	Rice mixed dishes	6.0	55.8
6	Pasta mixed dishes, excludes macaroni and cheese	5.7	61.6
7	Yeast breads	5.7	67.2
8	Burgers	4.3	71.5
9	Rice	4.2	75.7
10	Oatmeal	4.1	79.8
**Other Hispanic**			
1	Rice	16.4	16.4
2	Rice mixed dishes	13.0	29.4
3	Pizza	9.7	39.1
4	Yeast breads	8.9	48.0
5	Pasta mixed dishes	7.1	55.1
6	Other Mexican mixed dishes	5.8	60.8
7	Tortillas	4.8	65.6
8	Oatmeal	4.6	70.2.
9	Burgers	4.3	74.5
10	Egg/breakfast sandwiches	3.4	77.9
**White**			
1	Pasta mixed dishes	13.8	13.8
2	Pizza	11.7	25.4
3	Yeast breads	10.4	35.8
4	Burritos and tacos	9.6	45.4
5	Oatmeal	4.9	50.3
6	Burgers	4.4	54.7
7	Rolls and buns	4.3	59.0
8	Macaroni and cheese	3.5	62.6
9	Rice mixed dishes	3.5	66.0
10	Rice	3.1	69.1
**Black**			
1	Pasta mixed dishes	13.2	13.2
2	Pizza	9.3	22.5
3	Yeast breads	8.7	31.2
4	Rice	7.5	38.7
5	Burgers	6.3	45.0
6	Rice mixed dishes	6.3	51.2
7	Macaroni and cheese	5.6	56.9
8	Grits and other cooked cereals	5.0	61.9
9	Oatmeal	4.6	66.5
10	Burritos and tacos	3.8	70.3
**Asian American**			
1	Rice	37.3	37.3
2	Yeast breads	9.1	46.4
3	Fried rice and lo/chow mein	7.1	53.5
4	Pasta mixed dishes	6.5	60.0
5	Oatmeal	5.9	66.0
6	Rice mixed dishes	5.6	71.6
7	Pizza	4.9	76.5
8	Egg rolls, dumplings, sushi	3.9	80.3
9	Pasta, noodles, cooked grains	3.1	83.4
10	Burritos and tacos	2.8	86.2

Abbreviation: NHANES, National Health and Nutrition Examination Survey.

a National Health and Nutrition Examination Survey 2011–2016 (21).

b Cumulative percentage is the running total of percentage values. For a given source of sugar, the cumulative percentage represents the combined percentage of added sugars from that source and all sources with a higher ranking. Only the top 10 sources are shown; therefore, cumulative percentage does not add to 100%.

Asian American children consumed a greater percentage of their calories from high GI foods compared with all other races/ethnicities (*P* < .05), even when accounting for US nativity and poverty index. On average, Asian American children consumed more than one-third (mean, 34.8%; SD, 22.6) of their calories from high GI foods, whereas White children consumed about 31% of their calories from high GI foods (mean, 31.0%; SD, 13.1) (Figure).

Asian American children consumed greater quantities of high GI foods (*P* < .001), even when accounting for US nativity and poverty. On average, 23.8% (SD, 17.62) of total grams consumed in a day were from high-GI foods among Asian American children, whereas 18.8% (SD, 9.9) of total grams consumed in a day were from high GI foods among White children.

The top type of high-GI food was pizza among White, Black, other Hispanic, and other race/ethnicities. Rice was the top type among Asian American children, and burritos and tacos were the top type among Mexican American children. Among Asian Americans, rice accounted for 31% of total high GI foods consumed, or 44% if fried rice and mixed rice dishes were included. Across other race/ethnicities, the top sources accounted for 14% to 17% of total high-GI foods consumed. We also calculated the top 10 types of high GI foods consumed by race/ethnicity (Table 3).

**Table 3:** Ranked Population Proportion of 10 High Glycemic Index Foods Consumed Among Children, Aged 6–11 (N = 3,112), by Race and Ethnicity, NHANES 2011–2016^a^

Race/Ethnicity, Rank	Food Source	Percentage	Cumulative Percentage^b^
**Mexican American**			
1	Candy not containing chocolate	14.4	14.4
2	Ready-to-eat cereal, higher sugar	12.9	27.4
3	Cookies and brownies	9.5	36.9
4	Jams, syrups, toppings	7.0	43.9
5	Sugars and honey	6.4	50.2
6	Candy containing chocolate	5.9	56.1
7	Soft drinks	5.6	61.7
8	Tomato-based condiments	5.3	67.0
9	Sweetened fruit drinks	3.9	70.9
10	Cakes and pies	3.1	73.9
**Other Hispanic**			
1	Ready-to-eat cereal, higher sugar	11.8	11.8
2	Candy not containing chocolate	10.4	22.2
3	Jams, syrups, toppings	9.2	31.4
4	Cookies and brownies	8.5	40.0
5	Sugars and honey	8.3	48.3
6	Candy containing chocolate	5.8	54.1
7	Tomato-based condiments	4.7	58.8
8	Sweetened fruit drinks	4.2	62.9
9	Doughnuts, sweet rolls, pastries	4.0	67.0
10	Not included in a food category	4.0	70.9
**White**			
1	Candy not containing chocolate	18.8	18.8
2	Cookies and brownies	12.0	30.9
3	Jams, syrups, toppings	9.2	40.0
4	Ready-to-eat cereal, higher sugar	8.0	48.0
5	Sugars and honey	7.0	55.0
6	Candy containing chocolate	6.6	61.6
7	Tomato-based condiments	4.2	65.8
8	Ice cream and frozen dairy desserts	3.5	69.3
9	Doughnuts, sweet rolls, pastries	3.2	72.5
10	Soft drinks	3.1	75.6
**Black**			
1	Candy not containing chocolate	14.9	14.9
2	Jams, syrups, toppings	11.4	26.3
3	Ready-to-eat cereal, higher sugar	11.2	37.5
4	Cookies and brownies	10.8	48.3
5	Sugars and honey	7.6	55.9
6	Tomato-based condiments	6.3	62.1
7	Sweetened fruit drinks	6.3	68.4
8	Doughnuts, sweet rolls, pastries	3.5	71.9
9	Soft drinks	3.2	75.1
10	Cakes and pies	2.8	77.9
**Asian American**			
1	Candy not containing chocolate	12.6	12.6
2	Sugars and honey	12.0	24.6
3	Jams, syrups, toppings	10.4	35.0
4	Cookies and brownies	10.3	45.3
5	Ready-to-eat cereal, higher sugar	9.6	54.9
6	Candy containing chocolate	8.7	63.6
7	Ice cream and frozen dairy desserts	4.0	67.5
8	Tomato-based condiments	3.4	70.9
9	Cakes and pies	2.9	73.9
10	Sweetened fruit drinks	2.3	76.2
**Other, Non-Hispanic**			
1	Candy not containing chocolate	14.3	14.3
2	Ready-to-eat cereal, higher sugar	11.4	25.7
3	Jams, syrups, toppings	11.3	36.9
4	Candy containing chocolate	8.0	44.9
5	Cookies and brownies	7.6	52.5
6	Sugars and honey	7.1	59.6
7	Cakes and pies	4.6	64.2
8	Sweetened fruit drinks	3.8	68.0
9	Ice cream and frozen dairy desserts	3.5	71.5
10	Tomato-based condiments	3.3	74.8

Abbreviation: NHANES, National Health and Nutrition Examination Survey.

a National Health and Nutrition Examination Survey 2011–2016 (21).

b Cumulative percentage is the running total of percentage values. For a given source of sugar, the cumulative percentage represents the combined percentage of added sugars from that source and all sources with a higher ranking. Only the top 10 sources are shown; therefore, cumulative percentages do not add to 100%.

## Discussion

We found some similarities in the top sources of added sugars in children’s diets among the races/ethnicities studied. Candy and cereals contributed substantially to added sugar intake, although some differences by race/ethnicity were noted. Soft drinks did not contribute as much added sugar intake for Asian American children, although they were a top source for many other races/ethnicities. Rice was a popular high-GI food across multiple racial/ethnic groups. High-GI foods were more prevalent in the diets of Asian American and other Hispanic children, groups disproportionately burdened by nonalcoholic fatty liver disease.

Breakfast foods (eg, cereal, baked goods) were major sources of added sugar. Cereal appeared within the top 5 sources across all races/ethnicities. A 2014 analysis of cold cereals sold in the United States found that 92% of cereals had added sugar, and that all cereals that were marketed to children contained added sugar. On average, children’s cereals have 40% more sugar than cereals marketed to adults and may contain more sugar than cookies (22). Television advertising of high-sugar breakfast cereals that is directed at children has been positively associated with higher intake of high-sugar cereals (23). Our findings support recommendations and efforts to restrict child-directed marketing to limit excessive sugar consumption.

Soft drinks did not contribute to added sugar intake among Asian Americans in high quantities or similar amounts to children of other race/ethnicities. Current initiatives aimed at reducing added sugar intake have focused on taxing sugar-sweetened beverages, specifically soft drinks. Given our findings, such taxes might not be effective with Asian Americans. Focusing on reducing sugary drinks is imperative given the prevalence of their consumption and resultant adverse health effects (24), but additional efforts should be made to target other unhealthy dietary behaviors that contribute to added sugar consumption and to focus on foods, shifting the conversation from single nutrients to dietary patterns.

In 2016, the US Food and Drug Administration established that added sugar content would be included on the Nutrition Facts Panel (NFP) of products to call attention to hidden sugars in foods (eg, cereals) (25). A study conducted before the final ruling aimed to measure whether the proposed changes to the panel would influence young adults’ purchase intentions. Although many of that study’s participants viewed the added sugars component on the panel, participants exhibited no changes in purchase intentions (25). Although changing nutrition labeling might not be enough to change consumer behavior, it might stimulate the industry to reformulate products. In 2003, the Food and Drug Administration required the disclosure of trans fat content on the Nutrition Facts panel, which led to industry reformulation of ingredients (26).

Rice was the leading type of high-GI food consumed by Asian Americans. Asian American children consumed more grams and calories of high-GI foods than did children of other races/ethnicities. Considering the link between diabetes, nonalcoholic fatty liver disease, and refined grains, greater consumption of high-GI foods might contribute to the high burden of those conditions among Asian Americans (17). Promotion of whole grain options, such as brown rice, which has a lower GI than white rice, may help reduce cardiometabolic risk among Asian American populations. Most research has focused on using low-carbohydrate and low-GI diets as treatment; future research should investigate whether these diets are also effective at prevention. Furthermore, given low levels of physical activity among Asian American children (27) and a lack of emphasis on exercise stemming from traditional social norms, (28) future efforts should consider physical fitness as part of the lifestyle change that includes diet modification.

Our study was the first to evaluate sources of added sugar and high GI foods among Asian American children and other races/ethnicities. Previous studies that have assessed nutrient sources have not included Asian Americans (28). However, our study has some limitations. Results of interviews about diet may not represent actual intake; rather, they provide a cross-sectional view of dietary intake for 1 day. Despite this, numerous studies have used 1 day of dietary recall to assess sources of dietary nutrients (28,29). Sample size restrictions prevented us from reporting beyond the aggregate Asian American group in the NHANES data. In addition to differences in demographics among Asian Americans by ethnic subgroup and country of origin, large variability exists in cardiometabolic risk (30).

Because NHANES is a nationally representative sample, we believe our findings are largely generalizable to US children. However, previous studies that included Asian Americans reported data that were skewed to high-income and well-educated individuals. As such, results might not represent low-income and less-educated Asian Americans in the NHANES sample (31).

Sources of added sugars in children’s diets varied across racial/ethnic groups. Policies and programs to reduce sugar intake might help to reduce the risk of developing cardiometabolic health conditions and help reverse the negative health effects of the disease. Current initiatives to reduce excess sugar intake through soda taxes may not effectively reach Asian American children, considering the variation in consumption patterns observed in our study. Additional strategies that consider specific foods identified as top sources of added sugars for all races/ethnicities (eg, cereal) and refined grains are needed to more equitably reduce diet-related risk of cardiometabolic disease.
